# The Effects of Time‐Restricted Feeding on Handgrip Strength, Vigilance, and Perceived Anxiety and Depression in Older Adults: A Comparative Study Between Active and Sedentary Populations

**DOI:** 10.1002/hsr2.70692

**Published:** 2025-04-21

**Authors:** Mohamed Ali Boujelbane, Khaled Trabelsi, Haitham Jahrami, Achraf Ammar, Atef Salem, Mohamed Kerkeni, Amir Charfi, Omar Boukhris, Cain C. T. Clark, Rabih Roufayel, Nicola Luigi Bragazzi, Wolfgang I. Schöllhorn, Jordan M. Glenn, Hamdi Chtourou

**Affiliations:** ^1^ Department of Training and Movement Science, Institute of Sport Science Johannes Gutenberg‐University Mainz Mainz Germany; ^2^ High Institute of Sport and Physical Education University of Sfax Sfax Tunisia; ^3^ Physical Activity, Sport, and Health, National Observatory of Sport Tunis Tunisia; ^4^ Research Laboratory: Education, Motricité, Sport et Santé, EM2S, High Institute of Sport and Physical Education of Sfax University of Sfax Sfax Tunisia; ^5^ Department of Movement Sciences and Sports Training, School of Sport Science The University of Jordan Amman Jordan; ^6^ College of Medicine and Medical Sciences Arabian Gulf University Manama Kingdom of Bahrain; ^7^ Ministry of Health Manama Kingdom of Bahrain; ^8^ Research Laboratory, Molecular Bases of Human Pathology Faculty of Medicine of Sfax University of Sfax Sfax Tunisia; ^9^ SIESTA Research Group, School of Allied Health, Human Services and Sport La Trobe University Melbourne Australia; ^10^ Sport, Performance, and Nutrition Research Group, School of Allied Health, Human Services and Sport La Trobe University Melbourne Australia; ^11^ College of Life Sciences Birmingham City University Birmingham UK; ^12^ College of Engineering and Technology American University of the Middle East Kuwait; ^13^ Laboratory for Industrial and Applied Mathematics, Department of Mathematics and Statistics York University Toronto Ontario Canada; ^14^ Neurotrack Technologies Redwood City California USA; ^15^ Department of Health, Human Performance and Recreation, Exercise Science Research Center University of Arkansas Fayetteville Arkansas USA

**Keywords:** aging, fasting, mental health, physical activity, wakefulness

## Abstract

**Background and Aims:**

Ramadan intermittent fasting (RIF), a form of time‐restricted feeding, influences various physiological and psychological functions. However, its effects on older adults remain insufficiently understood. This study examined the impact of RIF on physical and mental health parameters, comparing active and sedentary older individuals. Specifically, we assessed handgrip strength (HGS), vigilance performance, anxiety, and depression levels to determine whether regular physical activity mitigates potential adverse effects of RIF.

**Methods:**

Fifty‐eight older adults (mean age 62.93 ± 3.99 years; 50% female) participated in this study. They were classified into an active group (*n* = 26) and sedentary group (*n* = 32) based on self‐reported physical activity levels. Assessments were conducted before and during RIF and included HGS measurement using a handheld dynamometer, a digital psychomotor vigilance test, and validated questionnaires (General Anxiety Disorder‐7, Geriatric Depression Scale, and Physical Activity Scale for the Elderly).

**Results:**

During RIF, both groups showed significant improvements in vigilance, anxiety, and depression scores, with more pronounced benefits in the active group. However, sedentary participants experienced a decline in HGS, whereas active individuals maintained stable muscle strength.

**Conclusion:**

Regular physical activity during RIF appears to enhance vigilance and mental health while preventing muscle strength decline in older adults. These findings highlight the importance of maintaining an active lifestyle during RIF to support both physical and mental health in aging populations.

## 1 Introduction

Aging is a multifaceted process characterized by progressive biological, physiological, and mental changes, ultimately increasing vulnerability to health complications [1, 2, 3]. One of the most prevalent consequences of aging is the decline in muscle strength and physical function, which significantly impacts mobility, independence, and quality of life [4]. Simultaneously, aging is often accompanied by a deterioration in mental health, as reflected in elevated levels of stress, anxiety, and depression—core components of mental health [5, 6]. Poor mental health has been found to contribute to accelerated physical decline, leading to a higher risk of frailty, falls, and disability [7].

Given the strong interrelation between mental and physical health, increasing attention has been directed toward identifying physical biomarkers that can serve as indicators of mental health in aging populations [8].

Handgrip strength has been recognized as a valuable biomarker for assessing overall health status, including mental health [8]. Lower HGS values have been associated with higher levels of depression and anxiety, whereas stronger grip strength has been linked to greater emotional resilience and mental stability [9]. This association has been attributed to the role of neuromuscular efficiency and systemic inflammation, both of which are directly connected to numerous mental health outcomes [7]. Furthermore, HGS has been widely recognized as a significant predictor of long‐term health outcomes, including functional decline, frailty, and mortality risk in older individuals [10]. Given its ease of measurement and its ability to discern both physical and mental health, HGS has emerged as a critical tool for identifying individuals at an elevated risk of adverse health outcomes.

In addition to HGS, vigilance—defined as the capacity to sustain attention and to remain alert to stimuli over extended periods [11, 12] has been recognized as a cognitive function closely associated with mental health [13]. It is particularly sensitive to psychological states such as stress, anxiety, and depression. Moreover, vigilance constitutes a core component of executive functioning and plays a critical role in maintaining autonomy, functional capacity, and safety in everyday life, especially among older adults [14]. Despite its relevance, the influence of lifestyle‐related factors, such as physical activity (PA) and nutritional habits, on vigilance performance in aging populations warrants further investigation.

In an effort to mitigate the age‐related decline in muscle strength, cognitive function and mental health, several lifestyle interventions have been explored. Among these, intermittent fasting (IF) has gained substantial attention due to its potential to induce both metabolic and neurophysiological benefits [15]. One specific form of IF, religious intermittent fasting (RIF), is widely practiced for spiritual and religious reasons [16]. During RIF, healthy individuals abstain from food, drink, smoking, and other activities from dawn to sunset over a lunar month [17].

While several studies have examined the effects of RIF on metabolic health, its influence on mental health and functional status in aging populations remains controversial. Some findings suggest that RIF may alleviate symptoms of anxiety and depression, possibly due to its effects on stress regulation, circadian rhythms, and hormonal balance [18, 19]. However, other studies have reported no significant improvements in mental well‐being following RIF [20]. Similarly, research on vigilance during RIF has yielded inconsistent results, particularly in middle‐aged healthy adults [21, 22]. However, no studies have specifically examined its effects on vigilance in elderly populations, underscoring the need for further research. Furthermore, the impact of RIF on HGS has yet to be fully elucidated. While some evidence suggests that RIF does not significantly affect muscle strength in older individuals [23], the extent to which fasting‐induced changes in mental health may influence or mediate grip strength remains unclear. Given the strong link between mental health and muscular performance [24], further research is warranted to explore whether RIF may modulate HGS through changes in anxiety and depression levels.

Beyond fasting, PA has been widely recognized as a crucial factor for maintaining muscle strength and mental well‐being in older adults [25]. Regular PA has been shown to preserve HGS values, enhance neuromuscular function, and reduce the risk of sarcopenia [25]. In addition to its well‐documented effects on muscle health, PA has been found to promote mental well‐being by reducing stress, anxiety, and depressive symptoms [26]. Furthermore, PA contributes to improved reaction time and psychomotor performance, highlighting its role in supporting both physical and cognitive function [25]. Despite these established benefits, limited research has explored the combined effects of RIF and PA on HGS as a biomarker of mental health in aging populations. Given that both fasting and exercise independently influence metabolic, hormonal, and neuromuscular processes, investigating their potential synergistic impact on HGS and psychological well‐being is essential for developing effective interventions for older adults.

Although various studies have examined the effects of RIF and PA separately, insufficient evidence exists regarding their combined influence on HGS as a biomarker of psychological health in aging populations. Addressing this gap is essential for understanding whether a structured lifestyle intervention incorporating both fasting and exercise could provide therapeutic benefits for mental well‐being and muscle strength in older individuals. The present study aimed to (i) investigate the effects of RIF on anxiety, depression, and vigilance performance for active and sedentary older adults from before to during RIF, (ii) to examine the influence of RIF on HGS measures for active and sedentary older adults from before to during RIF, and (iii) to evaluate the relationships between HGS measures and behavioral alertness during a psychomotor vigilance test in active and sedentary older adults.

We hypothesized that active older adults would have a greater HGS measure and vigilance performance, and lower anxiety and depression scores than their inactive counterparts, from before to during RIF. Moreover, we hypothesized positive associations between HGS measures and vigilance performance in both groups would exist.

## 2 Materials and Methods

### 2.1 Participants

**Figure 1:**
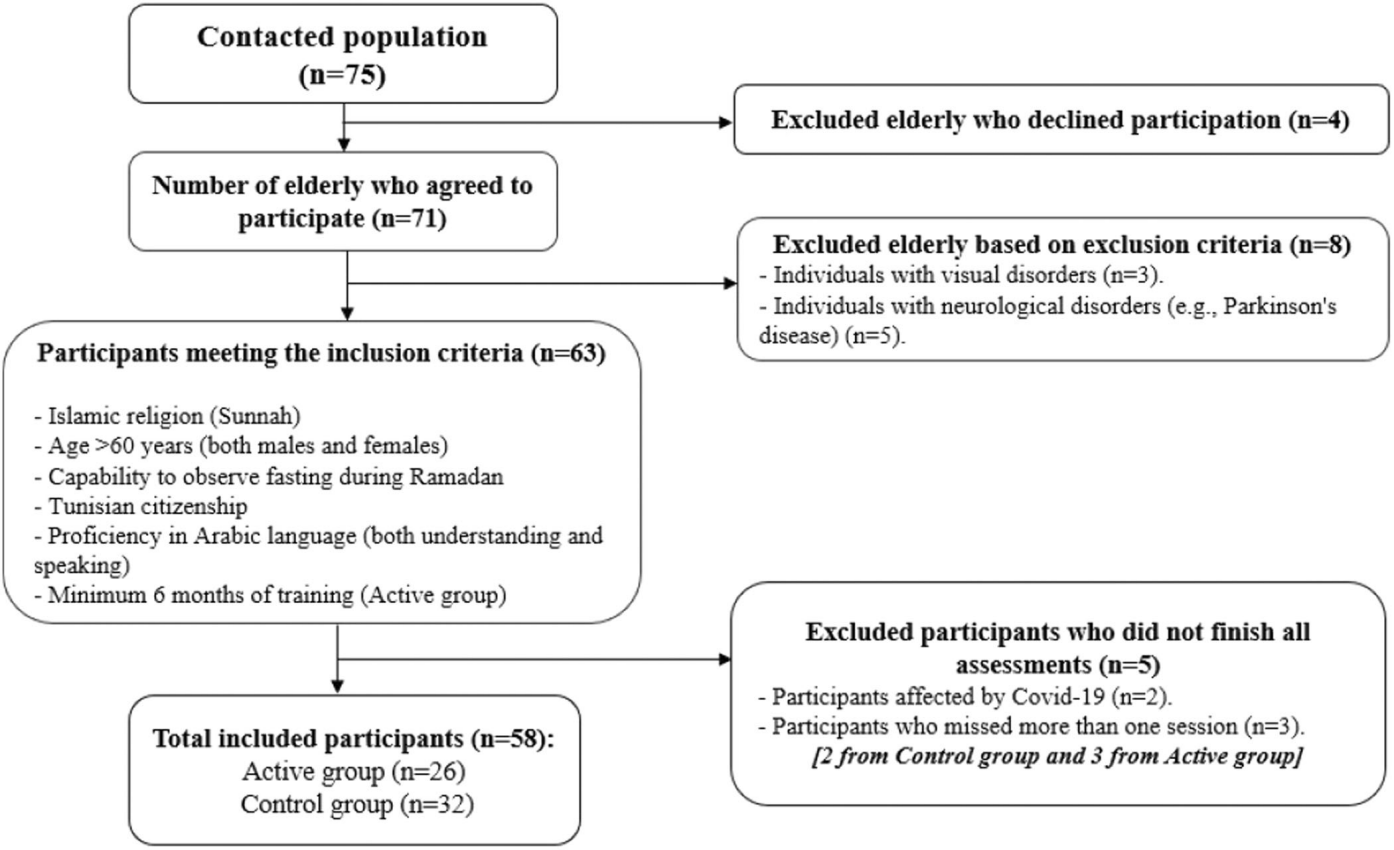
Flowchart illustrating the selection criteria and decision‐making process for elderly participants undergoing ongoing assessments.

Out of 75 contacted older adults, seventy‐one older adults expressed their willingness to participate in the present study (Figure 1). Eight subjects were excluded: three with visual disorders and five with neurological diseases (e.g., Parkinson disease). We identified 63 eligible older adults meeting the inclusion criteria: ≥ 60 years old, Islamic religion (*Sunnah*), having the Tunisian nationality, able to understand and speak Arabic language and able to fast during Ramadan. Active older people were required to have engaged in regular PA for at least 6 months before RIF. Due to their failure to complete all assessments, five participants were disqualified; two subjects contracted coronavirus disease 2019 (Covid‐19) and three subjects missed more than one training session. Therefore, 58 right‐hand dominant older people aged ≥ 60 years (62.9 ± 4.0 years; 50% female) were included and categorized into two groups: control group (*n* = 32) who did not follow any training program before and during Ramadan, and active group (*n* = 26), who were engaged in at least 6 months of training before Ramadan and continued their respective training program while observing Ramadan. The characteristics of the participants are shown in Table 1. Some individuals had cardiovascular and/or chronic diseases. However, they were allowed to participate in the training program and/or fast during Ramadan without posing any danger to their health, per their physician's approval. The training program's compliance rate was 93.59%. To be included in the study, each subject provided written consent in accordance with the Declaration of Helsinki. Subjects were aware that they could withdraw from the study at any time. This investigation was approved by the research ethics committee CPP SUD N°0322/2021.

**Table 1:** Demographic characteristics of the participants.

	Active group (*n* = 26)	Control group (*n* = 32)
Age (years) (Median [IQR])	61 [60; 63.5]	62 [60; 63.75]
BMI (kg/m²) (Median [IQR])		
Before‐R	26.13 [22.86; 27.06]	26.63 [20.07; 28.28]
During‐R	25.99 [22.6; 26.57]	26.37 [19.38; 28.63]
Sex [*N* (%)]		
Female	13 (50%)	18 (56.2%)
Male	13 (50%)	14 (43.8%)
Education attainment [*N* (%)]		
University	11 (42.3%)	7 (21.9%)
High school	9 (34.6%)	8 (25%)
Mild school	5 (19.2%)	12 (37.5%)
Primary school	1 (3.9%)	5 (15.6%)
Health status [*N* (%)]		
Healthy	14 (53.8%)	19 (59.4%)
Chronic disease	12 (46.2%)	13 (40.6%)
Type 2 diabetes	5 (19.2%)	6 (18.8%)
Dyslipidemia	1 (3.8%)	1 (3.1%)
Cardiovascular diseases	6 (23.1%)	6 (18.8%)
(mainly arterial hypertension)		

Abbreviations: BMI, body mass index; Before‐R, before Ramadan; During‐R, during Ramadan; IQR, Interquartile range; RIF, Ramadan fasting.

### 2.2 Experimental Design

The present study was conducted in Tunisia in spring 2022, during the Ramadan month, which lasted from 02 April to 02 May. Two assessment sessions were carried out in the laboratory: 2 weeks before Ramadan (Mean temperature = 15°C ± 2.1°C; Mean humidity= 76% ± 10.2%), and during the fourth week of Ramadan month (Mean temperature = 18°C ± 1.0°C; Mean humidity = 72% ± 8.3%). All sessions were conducted in the afternoon (4:00–6:00 p.m.). About 11‐12 h before their testing session during Ramadan, the participants consumed their last meal (i.e., *Suhur*) between 03:30 and 04:30 a.m. Physically active participants (i.e., active group) continued their physical training program while fasting throughout Ramadan at the same time as before (i.e., 5:30 p.m.), whereas the control group did not engage in any physical training activities. The physical training schedule encompassed three sessions per week of 1 h, interspersed with a recovery day. Each session began with 15 min warm up and finished with 10 min of stretching. The first session was based on team sports (i.e., volleyball or basketball), the second session included aquatic activities, while training circuits were carried out in the third session. Neither group experienced any fasting‐related side effects (e.g., headaches, hypoglycemia, or dizziness), thus reflecting the interventions feasibility. A similar evaluation process was performed by both groups, including an assessment of vigilance performance, based on validated digital psychomotor vigilance test [27]. Moreover, anxiety, depression, and physical and daily living activity were assessed with validated Arabic versions of the General Anxiety Disorder‐7 (GAD‐7) [28], the Geriatric depression scale (GDS) [29] and the physical activity scale for elderly (PASE‐A) [30, 31]. HGS measures were evaluated with a handheld dynamometer.

### 2.3 Psychomotor Vigilance Test (PVT)

PVT is a continuous 2‐min attention task used to assess vigilance, and response speed to visual stimuli. During the test, participants are required to click on a box as soon as red numbers appear at random intervals. False starts (i.e., response times < 100 msec) are excluded from the final analysis, and the average response time is considered as the target variable [27].

### 2.4 Handgrip Strength (HGS)

Muscle strength is measured using an analog handgrip with a calibrated dynamometer (T.K.K. 5401; Takei, Tokyo, Japan). Participants perform the test three times with both their nondominant and dominant hands, with a 30‐second rest period between assessments. HGS is determined by their maximum effort. To account for body mass influence, maximal HGS is standardized to the participants' body mass index (BMI) using the formula: Absolute handgrip strength (kg)/BMI (kg/m^2^) [32].

### 2.5 General Anxiety Disorder‐7 (GAD‐7)

The Arabic validated version of the GAD‐7 is used to evaluate anxiety levels. This seven‐item test assesses the severity of GAD symptoms over the last 2 weeks [33]. Scores range from 0 to 21, withcut‐points for mild, moderate, and severe anxiety at 5, 10, and 15, respectively [33].

### 2.6 Geriatric Depression Scale (Gds)

Depression levels are assessed using the Arabic validated GDS‐15 short version [29]. This 15‐item questionnaire prompts participants to respond yes or no to statements about how they felt over the previous week. Scores of 0–4 are considered normal, 5–8 indicate mild depression, 9–11 suggest moderate depression, and 12–15 indicate severe depression, depending on age, education, and complaints [34, 35].

### 2.7 Physical Activity Scale for the Elderly ‐ Arabic Version (PASE‐A)

The PASE is a validated and reliable instrument used in epidemiological studies to assess PA in older individuals [31]. It records the number of days per week and hours per day spent on various types of leisure activities. PASE scores are derived from the weighted frequency of 12 activity categories [30].

#### 2.7.1 Statistical Analysis

All statistical tests were processed using the statistical software STATISTICA (StatSoft, version 12, Paris, France). Values were presented as Mean ± SD for normally distributed data or as Median [IQR] for non‐normally distributed data. The following formula was used to determine the variation (Δ) recorded from before to during Ramadan session: Δ = During Ramadan – Before Ramadan. The normality of the data distribution was verified using the Shapiro–Wilk test. The baseline characteristics of the participants (presented in Table 1), including sex, educational attainment, and health status, were compared between the two groups using Pearson's chi‐squared test. The non‐parametric Mann–Whitney *U* test was performed as age and body mass index (BMI) were not distributed normally.

Handgrip nondominant scores were normally distributed across all tested variables. Therefore, a two‐way mixed analysis of variance (ANOVA) was used with two groups (Control vs. Active) and two Ramadan sessions (Before Ramadan vs. During Ramadan). Then, a pairwise comparison was conducted using a Bonferroni post hoc test if the ANOVA revealed a significant interaction. Non‐parametric tests were applied for other parameters for which normality could not be verified: The Mann–Whitney test was used to compare the means of the two groups before, during, and at the Δ level to analyze Ramadan × Group interaction, whereas the Wilcoxon test was used to compare each group's comparison from before to during Ramadan. The significance for all analyses was set at *p* < 0.05. Effect sizes (ES) defined as partial eta‐squared *ηp*² for the ANOVA Group × Ramadan interaction, were used to assess the magnitude of significant findings [36]. The corresponding *ηp*² values for small, moderate, and large ES were demarcated at 0.01, 0.06, and 0.14, respectively [36]. Moreover, non‐parametric partial correlation coefficients (i.e., Spearman's *ρ* test) were computed to investigate possible relationships between HGS and nHGS nondominant and dominant, and vigilance performance. The following assessment was given to the partial correlation coefficient *r*: < 0.19: no correlation; ≥ 0.20 to ≤ 0.39: low correlation; ≥ 0.40 to ≤ 0.59: moderate correlation; ≥ 0.60 to ≤ 0.79: moderately high correlation; ≥ 0.8: high correlation [37].

## 3 Results

Regarding the participants' initial characteristics (Table 1), there were no significant differences between active and inactive participants.

The main results recorded before and during Ramadan are presented in Table 2. The two‐way ANOVA (Ramadan × group) for handgrip nondominant scores revealed no significant Ramadan × group interaction (*F*_(1,56)_ = 0.102; *p* = 0.750; *ηp*² = 0.001). Independent samples *t*‐test revealed no significant differences between groups at any time period. Paired samples *t*‐test showed that handgrip nondominant scores decreased significantly from before to during Ramadan in the sedentary group (*p* = 0.001), with no change in the active group (*p* = 0.132).

**Table 2:** Main findings before and during Ramadan for active and sedentary groups.

Parameters	Groups	During‐R vs. Before‐R			Active vs. Control groups					
		Median [IQR]/Means ± SD			Before‐R		During‐R		Δ	
		Before‐R	During‐R	Δ	*T* test/Mann–Whitney	*p*‐value	*T* test/Mann– Whitney	*p*‐value	*T* test/Mann– Whitney	*p*‐value
Total GDS score (A.U)	Active group	4.5 [7; 2]	3.5 [5; 2]a	−1 [0; −4]	*z* = −0.262	0.794	*z* = −0.613	0.540	*z* = −0.291	0.771
	Control group	5 [5.75; 2]	4 [5.75; 2]a	−1 [0; −3]						
Total GAD‐7 score (A.U)	Active group	6 [9; 3]	3 [5.75; 1]a	−3 [−2.25; −12]	*z* = −0.557	0.578	z = −1.377	0.168	z = −0.739	0.460
	Control group	8 [10.75; 1]	4.5 [7; 0]a	−3 [0; −10]						
HGS nondominant (kg)	Active group	24.87 ± 7.90	24.46 ± 7.10	−0.40 ± 1.32	*t* = 0.086	0.932	*t* = 0.589	0.559	*t* = 1.807	0.067
	Control group	24.71 ± 6.17	23.52 ± 5.04a	−1.18 ± 1.85						
HGS dominant (kg)	Active group	27.3 [31.18; 12.3]	27.4 [30.63; 13.4]	−0.4 [0.35; −2.1]b	*z* = −0.039	0.969	*z* = −0. 650	0.650	*z* = −2.833	0.005
	Control group	27.3 [30; 16.5]	25.55 [29.75; 16.9]a	−1.05 [−0.42; −3.6]						
nHGS non ‐dominant (A.U)	Active group	0.93 [1.21; 0.47]	0.91 [1.18; 0.56]	0 [0.02; −0.12]b	*z* = −0.313	0.755	*z* = −0. 797	0.425	*z* = −2.549	0.011
	Control group	0.95 [1.09; 0.39]	0.9 [1.03; 0.47]a	−0.04 [0; −0.21]						
nHGS dominant (A.U)	Active group	1.05 [1.2; 0.48]	1.06 [1.21; 0.56]	0 [0.02; −0.08]b	*z* = −0.188	0.851	*z* = −0.813	0.416	*z* = −2.924	0.003
	Control group	0.97 [1.16; 0.56]	0.94 [1.09; 0.57]a	−0.03 [0; −0.14]						
Vigilance response time (msec)	Active group	486 [514.75; 355]	340 [403.25; 277]ab	−94.5 [−63.5; −344]b	*z* = −0.524	0.600	z = −4.465	< 0.001	*z* = −2.056	0.040
	Control group	463 [611; 386]	450 [507.5; 358]a	−64 [20.75; −315]						
PASE‐A scores (A.U)	Active group	150.31 [159.7; 143.8]b	123.02 [127.62; 100.89]ab	−36.85 [−17.63; −62.15]b	*z* = −6.496	*p* < 0.001	*z* = −6.301	*p* < 0.001	*z* = 2.447	0.014
	Control group	97.98 [121.78; 64.5]	79.72 [99.43; 63.12]a	−22.08 [−14.72; −28.63]						

Abbreviations: A.U, arbitrary unit; Before‐R, before Ramadan; During‐R, during Ramadan; Δ, difference between during and before Ramadan; GAD‐7, General Anxiety Disorder‐7; GDS, Geriatric Depression Scale; HGS nondominant = handgrip strength nondominant; HGS dominant, handgrip strength dominant; IQR, Interquartile range; kg, Kilogram; msec, millisecond; nHGS, nondominant normalized handgrip strength nondominant; nHGS dominant, normalized handgrip strength dominant; PASE‐A, physical activity scale for elderly in Arabic version.

a. significantly different from before Ramadan at *p* < 0.05.

b. significantly different compared to control group at *p* < 0.05.

For the handgrip dominant values, the Mann–Whitney *U* test showed a significant difference between the two groups at Δ level (*z* = −2.833; *p* = 0.005), with a more pronounced decrease in the sedentary group (Δ HGS = −1.05 [−0.42; −3.6]) compared to active participants (Δ HGS = −0.4 [0.35; −2.1]). The Wilcoxon test showed HGS dominant decreased significantly in the sedentary group (*p* < 0.001), while there was no change from before to during Ramadan for the active group (*p* = 0.101).

Concerning the nHGS nondominant and dominant scores, no changes were recorded from before to during Ramadan for the active group whereas a significant decrease (*z* = −3.029, *p* = 0.002; *z* = −3.328, *p* = 0.001, respectively) were shown in sedentary group.

For vigilance performance, significant differences were observed in responses time between the two groups during Ramadan (*z* = −4.465, *p* < 0.001) and at Δ level (*z* = −2.056, *p* = 0.040), whilst no difference was observed before Ramadan. A significant increase of performance (i.e., lower responses time scores) from before to during Ramadan in reaction time was shown in the active (*p* < 0.001) and sedentary groups (*p* = 0.039), with a higher increase of performance in active compared to sedentary group (Δ response time = −94.5 [−63.5; −344] vs. −64 [20.75; −315], respectively).

A significant improvement was recorded from before to during Ramadan in the GAD‐7 and GDS scores for active (*p* = 0.003; *p* = 0.010) and sedentary (*p* = 0.021; *p* = 0.010) older adults, with no significant difference between groups.

PASE‐A scores differed significantly between the two groups before Ramadan (*p* < 0.001; *r* = 0.853), during Ramadan (*p* < 0.001; *r* = 0.827), and at Δ level (0.014; *r* = 0.321), with the lower values in the sedentary compared to the active group. Moreover, a significant decrease was shown between pre to during Ramadan in active (*p* < 0.001; *r* = 0.874) and sedentary (*p* < 0.001; *r* = 0.803) groups.

Spearman's *ρ* test revealed no significant correlation between HGS and nHGS nondominant and dominant, and vigilance performance for both groups (Supporting Information S1: Tables S1 and S2). Moreover, no other significant correlations were found between different studied variables.

## 4 Discussion

This study represents a pioneering effort to assess the effects of RIF on anxiety, depression, vigilance performance, and HGS measures for active and sedentary older adults.

This study represents a pioneering effort to assess the effects of RIF on anxiety, depression, vigilance performance, and HGS measures for active and sedentary older adults. Before RIF, no significant differences were recorded in baseline scores for all parameters, except PASE‐A scores, which could be explained by the physical training program started at least 6 months before the study in active group. During RIF, significant differences were shown in vigilance performance and PASE‐A scores with greater values for physically active older adults. The two groups differed significantly at Δ level in PASE‐A values, vigilance scores, HGS dominant, and nHGS nondominant and dominant, with greater performance in the active group. From before to during RIF, the sedentary group decreased significantly in HGS nondominant and dominant, and nHGS nondominant and dominant, whereas no change was shown in the active group for HGS and nHGS measures. Contrarywise, both groups revealed a significant increase in vigilance performance, whilst a significant improvement was recorded in GAD‐7 and GDS scores, respectively, for active and inactive older adults. However, we found no significant correlations between vigilance response time scores, and HGS and nHGS measures.

Single studies revealed no effects of intermittent fasting on HGS measures in untrained adults [38, 39]. However, Bigard et al. [40] showed a decrease in muscular force in senior pilots during RIF, similar to present findings, in sedentary older adults. These findings may be attributed to reduced muscle protein synthesis, primarily caused by inadequate dietary protein intake, leading to potential alterations in the excitation‐contraction coupling mechanism and the type II muscle fiber architecture crucial for anaerobic metabolism [41]. Regarding the active older participants, no changes were recorded from pre to during RIF in any HGS measures. These results are in line with the findings of Bouhlel et al. [42], also indicating a lack of significant alterations in HGS among trained men during RIF. The present findings highlight the potential effects of a continuous combined physical program that may have counteracted some harmful effects related to the inadequate dietary protein intake, maintaining muscular strength in physically active participants by positively influencing the type I and type II muscle fiber size [43]. Nonetheless, to avoid making decisions based on the often erroneously applied democratic principle of the majority, future studies on individual hormonal changes that depend on circadian rhythms and nutritional habits are warranted to bring more enlightenment in the understanding of these phenomena. Furthermore, our findings revealed a significant improvement in vigilance performance and mood status (i.e., less depression and anxiety) for both groups from before to during RIF. These results support the previous hypothesis that almost any form of fasting could be accompanied by greater alertness and subjective sensations of well‐being, mood improvement, and, sometimes, feelings of euphoria [44, 45]. The enhancement in PVT scores in the two groups may be explained by a greater excitation of their respective dominant cerebral hemispheres [46]. In the case of right‐handed individuals, the left hemisphere is usually the dominant side, responsible for overseeing language, speech, and fine motor skills. Consequently, during cognitive tasks like the PVT, the dominant hemisphere may play a significant role, leading to better performance [46]. This suggests that RIF positively influences the dominant areas of activation, predominantly the inferior parietal lobule and posterior parietal cortices that are dominant in the control of vigilant attention, more particularly, in a positive emotional situation devoid of sadness and anxiety‐depressive disorders [46]. Furthermore, physically active individuals showed greater improvements in sustained vigilant attention than sedentary individuals, suggesting a positive effect of PA program adherence on vigilance performance [47] via the enhancement of brain‐derived neurotrophic factor expression in the frontal cortex [48], with higher concentration in the dominant hemisphere compared to the nondominant [49]. This may explain the greater percent of increase in performance (i.e., lower response time scores) recorded in the active group, compared to the sedentary group, due to the combined effects of RIF and PA on the dominant hemisphere [50].

Furthermore, our study revealed RIF can have a beneficial impact on psychological factors, including stress and anxiety, for both groups, with greater scores in the active group, from before to during RIF. This suggests Ramadan may be associated with several lifestyle changes, besides fasting, such as having a spiritual dimension (i.e., Quran reading groups, and *Attarawih* prayers) and a social component (i.e., shopping, and social meetings), which may be absent from other types of fasting and may be beneficial for preventing depression and anxiety [51]. Whether the findings are moderated by cultural mindsets, for example, religions, has to be shown in more differentiated and interdisciplinary studies of individuals from various religions and/or nonreligious backgrounds. Because regular fasting is a crucial constituent of many cultures in their origins, the common and primary intention for people's health is at hand.

Tobacco abstinence, for instance, has been linked to reduced depressed symptoms during RIF [52]. Moreover, waking up for the predawn meal (i.e., *Suhur*) and dawn prayer in Ramadan period might shorten the sleep period for Muslim fasters. According to some studies, reduction in depressive symptoms is also linked to acute reduced sleep duration [53, 54]. However, a recent meta‐analysis by [55], employing a methodologically rigorous approach, did not find conclusive evidence supporting the association between sleep deprivation and depressive outcomes. Therefore, investigating both main and side effects of extended sleep reduction, with a focus on recording daytime sleeping behavior is warranted. In this context, sleeping behavior during the day should be documented in future studies as well (actigraph and vigilance). These results are in line with previous studies revealing the potential effects of RIF on neuropsychological parameters, including anxiety and depressive symptoms [18, 56, 57]. Independent of cultural habits, regular PA in older individuals can help to reduce levels of anxiety and depression [26], suggesting older adults who engage in PA report higher levels of self‐esteem, which helps with psychosocial growth and emotional balance [58].

Although vigilance was preserved, and even improved, due to the effect of RIF on the dominant hemisphere in both groups, HGS recorded a slight decrease in the active group and was significantly altered in the control group, suggesting that this decrease may not be related to the cognitive function decline but instead linked to an impairment of daily living activity score manifested by the decrease in PASE scores accentuated in sedentary subjects. This could explain the lack of associations between HGS measures and cognitive function (i.e., alertness) in both groups. Our results are in accordance with those of other studies revealing no association between HGS and nHGS nondominant and dominant, and executive function performance, including vigilance and sustained attention [32, 59].

### 4.1 Limitations, Strengths, and Future Studies

Despite the novelty of our study, some limitations should be acknowledged and addressed in future studies. Coaches of the elderly individuals focused on maintaining the same duration and intensity in training sessions throughout the Ramadan month, as before RIF. Nevertheless, the reduction in PASE‐A scores in both groups could not be attributed to the decline in PA level, particularly for the active group. This decrease in energy expenditure may be due to a change in daily habits (e.g., gardening, home organization). As such, it is advised to increase/maintain these everyday activities to regular frequencies. We advocate that future research should therefore evaluate and control these confounding factors. Despite the existence of numerous studies in the senior population confirming the strong association between hydration status, nutrition, and cognitive performance [60, 61], as well as between dietary intake and muscular strength [62], we did not investigate hydration status, and nutritional intake before and during RIF, as well as in predawn meal, and breakfast. Further investigations of these variables before and during RIF are needed. Furthermore, the absence of precise measurement of individual medication dosages among patients with chronic diseases in both active and sedentary groups represent another limitation in our study. However, despite the lack of detailed dosage information, it is important to note there was no observed difference in medication consumption profiles between the two groups. Strengths of this study include its pioneering approach to evaluating the effects of RIF on both physical and psychological parameters in older adults, as well as its inclusion of both active and sedentary participants. This design provides valuable insights into the potential benefits of PA in mitigating some of the adverse effects associated with fasting. Additionally, the study ensured that the number of participants with chronic diseases and their medication usage profiles were comparable between the two groups. This balance minimizes the risk of these factors disproportionately influencing the results, thereby enhancing the reliability of the findings.

For future studies, we recommend that researchers from countries practicing RIF explore the psychological impact of fasting across diverse cultural contexts. Such investigations could yield valuable insights, providing a broader perspective and enhancing the generalizability of findings. Additionally, future research should focus on the role of hydration, nutrition, and sleep patterns during RIF, as well as the long‐term effects of fasting on physical and cognitive health in older adults. Investigating the impacts of several other cultural traditions using neurophysiological measurements is also warranted to identify possible underlying mechanisms. These areas of inquiry are critical for developing a more comprehensive understanding of the multifaceted impacts of RIF on health and well‐being.

## 5 Conclusion

Our findings indicate that RIF improves vigilance and reduces anxiety and depression in older adults, with greater benefits observed in those who are physically active. While HGS declined in sedentary individuals during RIF, it remained stable in the active group. These results suggest that regular, habitual PA during RIF helps preserve muscle strength and enhances mental health.

## Author Contributions

**Mohamed Ali Boujelbane:** conceptualization, methodology, formal analysis, writing – original draft, writing – review and editing, investigation, data curation. **Khaled Trabelsi:** methodology, supervision, writing – review and editing, project administration, conceptualization. **Haitham Jahrami:** methodology, writing – review and editing, conceptualization. **Achraf Ammar:** methodology, writing – review and editing, resources. **Atef Salem:** data curation, writing – review and editing, formal analysis. **Mohamed Kerkeni:** data curation, writing – review and editing, formal analysis. **Amir Charfi:** writing – review and editing, data curation, visualization. **Omar Boukhris:** writing – review and editing, formal analysis, validation. **Cain C. T. Clark:** writing – review and editing, visualization. **Rabih Roufayel:** writing – review and editing, visualization. **Nicola Luigi Bragazzi:** methodology, writing – review and editing. **Wolfgang I. Schöllhorn:** writing – review and editing. **Jordan M Glenn:** resources, software, writing – review and editing. **Hamdi Chtourou:** project administration; supervision, methodology, conceptualization, writing – review and editing.

## Ethics Statement

All participants provided written consent in compliance with the Declaration of Helsinki guidelines. Subjects were informed of their right to withdraw from the study at any time. This study was conducted under the approval of the research ethics committee CPP SUD N°0322/2021.

## Conflicts of Interest

According to the authors, the study was carried out without any financial or commercial ties that might be viewed as a potential competing interest.

## Transparency Statement

The lead author Achraf Ammar affirms that this manuscript is an honest, accurate, and transparent account of the study being reported; that no important aspects of the study have been omitted; and that any discrepancies from the study as planned (and, if relevant, registered) have been explained.

## Supporting information


**Table S1.** Correlations between HGS and nHGS non‐dominant and dominant measures, GDS and GAD7 scores, and vigilance performance for active group.
**Table S2.** Correlations between HGS and nHGS non‐dominant and dominant measures, GDS and GAD7 scores, and vigilance performance for control group.

## Data Availability

The data that support the findings of this study are available from the corresponding author upon reasonable request.
