# Supplementary material for: The Effects of Time‐Restricted Feeding on Handgrip Strength, Vigilance, and Perceived Anxiety and Depression in Older Adults: A Comparative Study Between Active and Sedentary Populations
Source: Health Sci Rep. 2025 Apr 21;8(4):e70692. doi: 10.1002/hsr2.70692 (PMC12010213; doi:10.1002/hsr2.70692)
Supplement: Supplementary file 1 — Table S1. Correlations between HGS and nHGS non‐dominant and dominant measures, GDS and GAD7 scores, and vigilance performance for active group. Table S2. Correlations between HGS and nHGS non‐dominant and dominant measures, GDS and GAD7 scores, and vigilance performance for control group. [file HSR2-8-e70692-s001.docx]

**Table S1.** Correlations between HGS and nHGS non-dominant and dominant measures, GDS and GAD7 scores, and vigilance performance for active group

| **Variables** | **Correlations** | | | | | | | |
| --- | --- | --- | --- | --- | --- | --- | --- | --- |
|  | HGS left  Before-R | HGS left  During-R | HGS right Before-R | HGS right During-R | nHGS left Before-R | nHGS left During-R | nHGS right  Before-R | nHGS right  During-R |
| GAD7 Before-R | 0.250 ns | 0.289 ns | 0.212 ns | 0.237 ns | 0.257 ns | 0.255 ns | 0.142 ns | 0.151 ns |
| GAD7 During-R | -0.050 ns | -0.074 ns | -0.037 ns | -0.021 ns | -0.055 ns | -0.127 ns | -0.131 ns | -0.112 ns |
| GDS Before-R | -0.115 ns | -0.114 ns | -0.154 ns | -0.126 ns | -0.111 ns | -0.116 ns | -0.195 ns | -0.172 ns |
| GDS During-R | 0.286 ns | 0.307 ns | 0.306 ns | 0.275 ns | 0.260 ns | 0.263 ns | 0.215 ns | 0.170 ns |
| Vigilance Before-R | -0.133 ns | -0.070 ns | 0.000 ns | -0.020 ns | -0.120 ns | -0.077 ns | -0.002 ns | -0.008 ns |
| Vigilance During-R | -0.138 ns | -0.117 ns | -0.157 ns | -0.146 ns | -0.111 ns | -0.089 ns | -0.111 ns | -0.080 ns |

*Note.* Abbreviations: Before-R = before Ramadan; During- R = during Ramadan; GAD-7 = General Anxiety Disorder-7; GDS = Geriatric depression scale; HGS non-dominant = Handgrip strength non-dominant; HGS dominant = Handgrip strength dominant; nHGS non-dominant = normalized handgrip strength non-dominant; nHGS dominant = normalized handgrip strength dominant; *** = p < 0.050; ** = p < 0.020; * = p < 0.001; ns = p>0.050

**Table S2.** Correlations between HGS and nHGS non-dominant and dominant measures, GDS and GAD7 scores, and vigilance performance for control group

| **Variables** | **Correlations** | | | | | | | |
| --- | --- | --- | --- | --- | --- | --- | --- | --- |
|  | HGS left  Before-R | HGS left  During-R | HGS right Before-R | HGS right During-R | nHGS left  Before-R | nHGS left During-R | nHGS right Before-R | nHGS right During-R |
| **GAD7 Before-R** | -0.145 ns | -0.048 ns | -0.081 ns | -0.091 ns | -0.178 ns | -0.086 ns | -0.149 ns | -0.142 ns |
| **GAD7 During-R** | -0.135 ns | -0.088 ns | -0.070 ns | -0.107 ns | -0.147 ns | -0.079 ns | -0.120 ns | -0.125 ns |
| **GDS Before-R** | -0.245 ns | -0.194 ns | -0.193 ns | -0.167 ns | -0.186 ns | -0.136 ns | -0.145 ns | -0.132 ns |
| **GDS During-R** | -0.057 ns | 0.012 ns | -0.011 ns | -0.005 ns | -0.047 ns | -0.011 ns | -0.000 ns | -0.023 ns |
| **Vigilance Before-R** | 0.185 ns | 0.142 ns | 0.082 ns | 0.149 ns | 0.130 ns | 0.059 ns | 0.023 ns | 0.038 ns |
| **Vigilance During-R** | 0.183 ns | 0.128 ns | 0.133 ns | 0.157 ns | 0.183 ns | 0.091 ns | 0.126 ns | 0.120 ns |

*Note.* Abbreviations: Before-R = before Ramadan; During- R = during Ramadan; GAD-7 = General Anxiety Disorder-7; GDS = Geriatric depression scale; HGS non-dominant = Handgrip strength non-dominant; HGS dominant = Handgrip strength dominant; nHGS non-dominant = normalized handgrip strength non-dominant; nHGS dominant = normalized handgrip strength dominant; ; *** = p < 0.050; ** = p < 0.020; * = p < 0.001; ns = p>0.050
